# ATP-related purinergic signaling via P2Y12 contributes to airway epithelial barrier integrity

**DOI:** 10.1186/s12860-026-00578-8

**Published:** 2026-02-25

**Authors:** Kota Tsuya, Shuichiro Maruoka, Shinichi Okamoto, Sotaro Shikano, Mari Ujike, Shiho Yamada, Yusuke Kurosawa, Kenji Mizumura, Yutaka Kozu, Yasuhiro Gon

**Affiliations:** Division of Respiratory Medicine, Department of Internal Medicine, Nihon University School of Medicine, 30-1 Oyaguchi-Kamicho, Itabashi-ku, Tokyo 173-8610 Japan

**Keywords:** Airway epithelial barrier, ATP, Purinergic receptors, Bronchial asthma, Tight junctions, Adherens junctions, P2Y12

## Abstract

**Background:**

The airway epithelial barrier, which defends against external pathogens, is compromised in bronchial asthma. This study investigated the roles of adenosine triphosphate (ATP) and specific purinergic receptors in airway epithelial barrier function. Using the 16HBE14o- human airway epithelial cell line, we measured barrier integrity using transepithelial electrical resistance and the apparent paracellular permeability coefficient.

**Results:**

ATP enhanced barrier integrity in a dose-dependent manner without affecting cell proliferation. Real-time PCR revealed an increased expression of tight and adherens junction proteins, including E-cadherin and occludin, following ATP stimulation. Our findings suggest that P2Y12 contributes to ATP-related purinergic signaling associated with enhanced airway epithelial barrier integrity.

**Conclusions:**

These findings suggest that ATP-related purinergic signaling via P2Y12 may contribute to the regulation of airway epithelial barrier integrity in vitro. Further studies in more physiologically relevant systems are warranted to determine the clinical relevance of these observations.

## Background

The airway epithelial barrier is a critical biological defense mechanism that prevents invasion by external pathogens and antigens. This barrier comprises adhesion structures that bridge the gaps between airway epithelial cells. These structures include tight junctions (TJs), which facilitate membrane attachment between adjacent cells, adherens junctions (AJs), which preserve cellular morphology, and desmosomes, which enable cellular connectivity [1–3]. TJs contain proteins such as claudin (CLDN), occludin, and zonula occludens, while AJs contain cadherin and other proteins. The expression levels of these proteins are intricately and dynamically regulated in response to external viral infections, house dust mite allergens (HDM), and internal mechanisms such as inflammation [4]. Notably, the airway epithelial barrier in patients with bronchial asthma is more susceptible to dysfunction than in healthy individuals, exacerbating disease progression [5–7].

Various factors contribute to the onset and development of bronchial asthma, including genetic predispositions, atopic conditions, airway hyperresponsiveness, low birth weight, and obesity, and environmental factors such as viral infections, mite allergens, smoking, air pollution, and diet. The interplay between these factors is crucial for the pathogenesis of asthma [8]. Environmental factors, particularly HDM, compromise the epithelial barrier through intrinsic proteases and trigger allergic inflammation via toll-like receptor 4, a critical innate immune receptor expressed in epithelial cells [9].

Additionally, environmental factors can exacerbate inflammation through mediators released at the site of airway inflammation, such as adenosine triphosphate (ATP), HAGM-1, and S100 proteins. Intriguingly, ATP plays a dual role; it is essential for cellular energy supply but also exerts distinct effects when released extracellularly, acting through purinergic receptors on the cell surface [10]. These receptors include the nucleotide-gated ion channel P2X and G-protein-coupled P2Y, where ATP is partially hydrolyzed to adenosine, which binds to its A1 receptor and primarily exerts an anti-inflammatory effect [11].

Endogenous factors such as uric acid, nucleic acids, and extracellular ATP, collectively termed alarmins, are released from damaged cells and are implicated in the pathogenesis of asthma. In mouse models using ovalbumin (OVA) as an antigen, elevated ATP levels in bronchoalveolar lavage fluid have been associated with OVA-induced allergic airway inflammation. Moreover, ATP inhibitors mitigate this inflammation and airway hyperresponsiveness, suggesting that OVA-induced inflammation is mediated through P2Y2 receptor activation [12].

Conversely, ATP also serves as a protective agent against inhaled antigens, infectious agents, and toxins by regulating ion channel permeability, promoting cellular migration, and stimulating mucus secretion through its receptors [13–15]. Despite the multifaceted impact of ATP on the airway epithelium, no studies have identified specific purinergic receptors that regulate the airway epithelial barrier function.

This study aimed to elucidate the role of ATP in modulating epithelial barrier function and to identify the specific P2 receptors involved. We assessed ATP-induced changes in airway epithelial barrier function using airway epithelial cell lines.

## Methods

### Cells and cell culture

The human airway epithelial cell line 16HBE14o- (16HBE) was obtained from Associate Professor Dieter C. Gruenert (Gene Therapy Center, Cardiovascular Research Institute, Department of Laboratory Medicine, University of California, CA, USA). 16HBE cells were cultured at 37 °C and in the presence of 5% CO_2_. Minimum essential medium (MEM; Sigma-Aldrich, Tokyo, Japan) supplemented with 10% fetal bovine serum (SAFC Biosciences, Lenexa, KS, USA) and a penicillin-streptomycin mixed solution (Nacalai Tesque, Inc., Kyoto, Japan) was used as the culture medium. In this study, 16HBE14o- cells were grown either as submerged monolayers on collagen-coated Transwell inserts or, in separate experiments, were differentiated under air–liquid interface conditions to more closely mimic the in vivo airway epithelium.

### Transepithelial electrical resistance (TER) measurement

To evaluate airway epithelial barrier function, TER was measured using the Millicell-Electrical Resistance System (Millipore Co., Bedford, MA, USA). A previous study reported a correlation between the barrier function of epithelial cells and the TER, which depends on the level of TJ formation in the epithelial cell layer [16]. TER was calculated using the following equation: (TER sample − TER blank) × surface area. TER values were expressed as Ω·cm² following the subtraction of the blank inserts’ resistance, and multiplying the resultant values by the surface area of the Transwell membrane.

### Apparent paracellular permeability coefficient (Papp) measurement

To evaluate the paracellular permeability of 16HBE, the Papp of monolayer cells in the Transwell was measured by the degree of diffusion of fluorescein isothiocyanate (FITC)-dextran (FD10s, Sigma-Aldrich, Tokyo, Japan). Transwells with 3.0-µm pore size and 6.5-mm diameter inserts were used. The FITC solution was dissolved in D-PBS(-) (Nacalai Tesque, Inc., Kyoto, Japan) to obtain a 10 mg/mL solution. The Papp measurements were performed under light-shielded conditions so that the transmittance would not be affected. Before the measurement, 20 µL of the medium in the Transwell apical chamber was removed, and 20 µL of FITC was added. After placing the wells in an incubator at 37 °C for 1 h, fluorescence was measured using a PTI fluorometer (excitation: 492 nm, emission: 520 nm). Papp was calculated using the following equation:

$${\rm{Papp (cm/s) = (dQ/dt)/(1/AC0)}}$$

dQ/dt: transmittance (µg/s), C0: initial concentration of FITC added to the upper layer of Transwell (µg/mL), A: Transwell surface area (cm).

### Measurement of barrier function upon ATP stimulation

The effect of ATP (#A2383-1G, Sigma-Aldrich, St. Louis, Missouri, USA) on the airway epithelial barrier function in 16HBE was examined. ATP solutions of different concentrations (125 µM, 250 µM, and 500 µM) were prepared by diluting with MEM before the stimulation of 16HBE. ATP concentrations of 125, 250, and 500 µM were selected as previous studies have demonstrated that epithelial surface liquids can reach ATP levels in the low-to-mid-hundreds of micromolars under mechanical or inflammatory stress, and that robust purinergic signaling in airway epithelia often requires similar ATP concentrations [13, 20].

The cells were stimulated after 24 h of culture, and TER and Papp were measured on the same day of stimulation (day 0). The no-stimulation group containing only culture media was used as the control (CTR), and the ATP stimulation groups (125 µM, 250 µM, and 500 µM) were compared with the CTR group. In addition, to confirm that the viable cell numbers do not affect the barrier function during measurement, the viable cell numbers in the CTR and ATP stimulation groups (500 µM) on days 0 and 3 were compared and examined. A 0.4% Trypan Blue solution (GibcoTM) was used for staining.

### Real-time PCR

We investigated whether the gene expression of epithelial barrier-related molecules changed upon ATP stimulation using real-time PCR. The cells were collected on day 3 after ATP stimulation. Total RNA was extracted from the collected samples using an Ambion Fast SYBR Green Cells-to-CT kit (Life Technologies, Carlsbad, CA, USA) according to the manufacturer’s protocol. Extracted RNA was reverse-transcribed into complementary DNA (cDNA). Real-time PCR was performed using the cDNA, Fast SYBR Green Master Mix (Applied Biosystems, Foster City, CA, USA), and gene-specific primers. The reaction was set up in a 96-well plate and run on the StepOne system (Applied Biosystems, Foster City, CA, USA). The thermal cycling conditions consisted of an initial denaturation step at 95 °C for 20 min, followed by 40 cycles of 3 s at 95 °C and 30 s at 60 °C. The relative gene expression levels were determined using the ΔΔCt method, with GAPDH as the internal control. A comparative quantification method was used to determine the amount of RNA present in each sample. Primers used for this assay are listed in Table 1. Relative mRNA expression levels were calculated in terms of ratios to the internal control gene and are presented as fold-changes normalized to the corresponding control samples.

**Table 1:** Specific primers for real-time PCR

Primers	Forward 5’→3’	Reverse 5’→3’
E-cadherin	AAGGTGACAGACGCCTCTGGATAGA	CATTCCCGTTGGATGACACACA
Occludin	CGAGGAGTGGGTTAAAAATGTGT	ACTGTCAACTCTTTCCACATAGTCAGA
ZO-1	CGGTCCTCTGAGCCTGTAAG	GGATCTACATGGGACGACAA
ZO-2	GAGGAGTAGGAGCAGGAGCA	GGAGCCAACTGACAGCTC
JAM-A	CCGTGTGGAGTGGAGTTTGA	TCACCCGGTCTCTATAGGAA
JAM-B	GCCTGCAAACCCCAAAGAA	GAGACACTCCGACCCAGTTT
JAM-C	TCAGCCCTTTATCGCTGTGA	GTACAGCCTTCGGCACTCTA
CLDN 1	GGATTTACTCCTATGCCGGCGACAACA	CTCTGGGACACGCAGGGACATCCA
CLDN 3	CATCACGTCGAGAACATCTG	TCGTACACCTTGCACTGCATCT
CLDN 4	AGCCTTCCAGGTCTCAACT	AGCAGCGAGTCGTACACCTT
CLDN 5	CCTGGACCACAACATCGTGA	AGCACCGAGTCGTACACTTT
CLDN 6	ATGCAGTGCAAGGTGTACGA	CCAGCAAGGTAGACCAGCAA
CLDN 7	AGGCATAATTTTCATCGTGG	GAGTTTGGACTTAGGGTAGAGCG
CLDN 9	ATGCAGTGCAAGGTGTACGA	TACACTGGGCACCTGTGATG
CLDN 11	CCCGGTGTGGCTAAGTACAG	GATTTGTGGGAGTCCATCCCC
CLDN 16	TGGGAATGCGTCAACAATGC	GGATGCTCCGCAAGTATGGA
CLDN 18	TACACATTTGGTGCGGCTCT	TAAAACGTCTGGTTGCAGCG
CLDN 19	GCATTGACAGGTGTGCTTGG	CAGCCCGACTCAGTGTGATC

### Reverse transcription PCR

Reverse transcription PCR was performed to confirm that the P2 receptor gene group was expressed on the surface of 16HBE cells. Purified RNA was reverse-transcribed into cDNA using a PrimeScript RT Master Mix (#RR036A; Takara Bio, Kyoto, Japan). PCR amplification of cDNA was performed using Amplitaq Gold DNA polymerase (Thermo Fisher Scientific, Waltham, MA, USA), GeneAmp 10x PCR Buffer (#JP6390, Applied Biosystems, Foster City, CA, USA), dNTP Mix (#0704071, AB, UK), and gene-specific primers. The thermal cycling conditions for the P2X receptors were an initial denaturation at 94 °C for 2 min, followed by 35 cycles of 30 s at 94 °C, 30 s at 60 °C, and 30 s at 72 °C. For P2Y receptors, the conditions were: 2 min at 94 ℃, followed by 40 cycles, each consisting of 30 s at 94 °C, 30 s at 62 °C, and 30 s at 72 °C. The amplified PCR products were visualized on a 1.8% agarose gel, stained with ethidium bromide, and observed under ultraviolet light. Primers used for this assay are listed in Table 2. No-template and minus-reverse transcriptase negative controls were included for each primer set in each experiment without yielding detectable bands, confirming the absence of contaminating genomic DNA or nonspecific amplification.

**Table 2:** Specific primers for reverse transcription PCR

Primers	Forward 5’→3’	Reverse 5’→3’
P2X1	GCTACGTGGTGCAAGAGTCA	GTAGTTGGTCCCGGTTCTCCA
P2X2	GCTCTTTCCCATCCATCGTG	GGAAGTGGAGGACCCCTGTAG
P2X3	ACAGCCGAGGGAAGAAGGAC	AGCCGGGTAGGAAGGTATTT
P2X4	GAGATTCCAGATGGCCAC	GACTTGAGTGGAATGATGG
P2X5	CTGTGCTGATGGGGTGTTCCT	CTGGGCTGGAATGAGCTAGT
P2X 6	ACTCTGTGTGGAGGGGACTG	GGCAAGTGGGTGCAGACT
P2X7	AAGCTGTGACCGCAGGGAAA	GCTCTTGGGCCTCTGTTTTG
P2Y1	AAAACCTAGCCCCTGACACT	GATCTGATGCCGGGATGACT
P2Y2	CCACCTGCCTTCCTCACTGC	TGGGAAATCTCAGGACTGG
P2Y4	CGTCTTCTGGCCTCCCTGCTCT	GCCCTGCACTCATCCCCTTTTCT
P2Y6	AGCTGGGGCATCAGGTTAAA	GCTGTAGGGGACCTGTAAG
P2Y11	CCTCTAGCCAGCCTCTATG	CACTGCGGCCCATGTAGATA
P2Y12	TTTGCCGCGAATTCATCACAC	ATTGGGGCACTTCAGGATAC
P2Y13	CCCCCTGGCTACCATGGAAAGA	TACAGAGGAGGGGGTGATG
P2Y14	TCTTTGGGGCCTACCATCGTTT	TCCGTCCCACTTCACTTTTC

### Measurement of barrier function using P2 receptor-selective agonists

To identify the P2 receptors involved in barrier function, P2 receptor-selective agonists were used. The following selective antagonists were used: αβ-MeATP (#M6517, Sigma-Aldrich, St. Louis, MO, USA), 2-MeSATP (#1062, TOCRIS Bioscience, Bristol, UK), adenosine diphosphate (ADP) (#A5285, Sigma-Aldrich, St. Louis, MO, USA), uridine diphosphate (UDP) (#3111, TOCRIS Bioscience), uridine triphosphate (UTP) (#94370, Sigma-Aldrich, St. Louis, MO, USA), and 2-MeSADP (#1624, TOCRIS Bioscience). Each agonist selectively stimulates the following receptors: αβ-MeATP stimulates P2 × 1, P2 × 2, P2 × 3, P2 × 4, P2 × 5, and P2 × 7; 2-MeSATP stimulates P2 × 1, P2 × 2, P2 × 3, P2 × 7, and P2Y13; ADP stimulates P2Y1, P2Y12, and P2Y13; UDP stimulates P2Y6 and P2Y14; UTP stimulates P2Y2, P2Y4, and P2Y6; and 2-MeSADP stimulates P2Y1, P2Y12, and P2Y13 (Table 3) [17]. For each agonist, the stimulation group (500 µM) was compared with the CTR group. 16HBE cells were cultured as a monolayer in 24-well Transwell inserts (Costar, New York, NY, USA), and the culture medium was replaced with a medium containing the reagents after 24 h. Reagents were added to both the apical and basolateral chambers of the Transwell, and TER and Papp were measured on day 3.

**Table 3:** P2 receptor classifications and their corresponding ligands

Receptor	Agonists	Antagonists
P2X1	ATP, 2-MeSATP, αβ-MeATP	—
P2X2	ATP, 2-MeSATP, αβ-MeATP	—
P2X3	ATP, 2-MeSATP, αβ-MeATP	—
P2X4	ATP, αβ-MeATP	—
P2X5	ATP, αβ-MeATP	—
P2X6	—	—
P2X7	ATP, 2-MeSATP, αβ-MeATP	—
P2Y1	ATP, ADP, 2-MeSADP	MRS2179
P2Y2	ATP, UTP	—
P2Y4	ATP, UTP	—
P2Y6	ATP, UTP, UDP	—
P2Y11	ATP	—
P2Y12	ADP, 2-MeSADP	AZD1283
P2Y13	ATP, ADP, 2-MeSADP, 2-MeSATP	MRS2211
P2Y14	UDP	—

### Measurement of barrier function using P2 receptor-selective antagonists

Next, TER was measured using selective antagonists of P2 receptors, which were found to affect the barrier function when selective agonists were used. The following selective antagonists were used: MRS2179 (#0900; TOCRIS Biosciences, Bristol, UK) and AZD1283 (#6085; TOCRIS Biosciences). MRS2179 blocks the P2Y1 receptor, AZD1283 blocks the P2Y12 receptor, and MRS2211 blocks the P2Y13 receptor. The TER values of ATP alone (500 µM), MRS2179, AZD1283, and MRS2211 (10 µM, 50 µM, and 100 µM) alone, and ATP + blocker administration groups were compared with those of the CTR group. The 16HBE cells were cultured as a monolayer in 24-well Transwell inserts (1 × 10^5^ cells/well), and the culture medium was replaced after 24 h. ATP was added after 15 min of pretreatment with culture medium containing the selective antagonist. ATP reagent was added to both the apical and basolateral chambers of the Transwell plate, and TER was measured on day 1.

### Preparation and transfection of small interfering RNA

To investigate whether ATP stimulation is involved in barrier function via P2Y receptors, we transfected specific short-interfering RNAs (siRNAs) into 16HBE cells. P2Y12-specific siRNAs were transfected into 16HBE cells using Lipofectamine RNAiMAX (#13778150, Thermo Fisher Scientific) according to the manufacturer’s protocol. Transfected cells were trypsinized and transferred to a Transwell chamber for 24 h. Reagents were added to both the apical and basolateral chambers of the Transwell, and TER and Papp were measured on day 1. The siRNAs used in transfection are listed below: Stealth RNAi siRNA Negative Control Med GC Duplex #2 (#12935-112, Thermo Fisher Scientific), P2RY12 siRNA (Thermo Fisher Scientific); Hs_P2RY12_7237.

### Statistical analysis

Data are presented as means with 95% confidence intervals. For comparisons between two groups, an unpaired t-test was used. For the evaluation of multiple groups, analysis of variance followed by Tukey’s post hoc test was used. Statistical significance was set at *P* < 0.05. All analyses were performed using GraphPad Prism 7 (La Jolla, CA, USA).

## Results

### ATP promotes airway epithelial barrier integrity

**Fig. 1:**
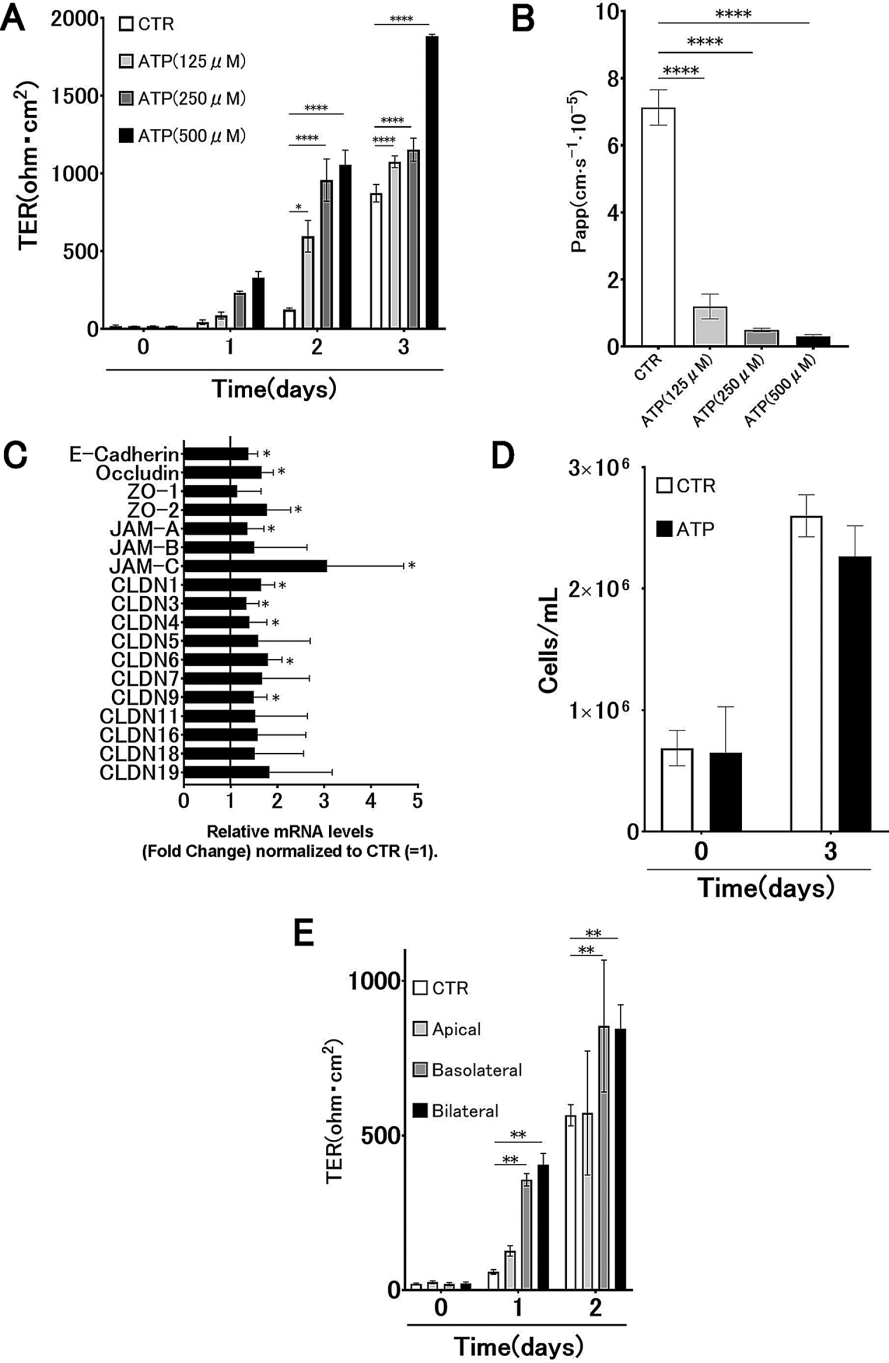
ATP promotes the formation of the airway epithelial barrier. (**A**) Changes in transepithelial electrical resistance (TER) over time following ATP stimulation (125, 250, and 500 µM) in 16HBE cells (*n* = 9). (**B**) Dextran permeability (Papp) after 3 days of ATP stimulation (*n* = 9). (**C**) Relative mRNA expression of tight junction- and adhesion-related genes following ATP stimulation. (**D**) Comparison of viable cell counts between control (CTR) and ATP-treated groups on days 0 and 3 (*n* = 6). No significant differences were observed (*P* = 0.98 and *P* = 0.27, Student’s t-test). (**E**) Changes in TER over time following ATP application to the apical, basolateral, or bilateral sides of 16HBE cells (*n* = 3). Results are presented as mean ± standard deviation. Statistical significance was assessed using Student’s t-test or analysis of variance as appropriate. *P < 0.05, **P < 0.01, ****P < 0.0005. Asterisks indicate significant differences compared with the CTR group at the corresponding time point

To determine the effect of ATP on airway epithelial barrier function, we measured TER values upon ATP stimulation. The TER values increased considerably in a dose-dependent manner (compared each day) on days 1, 2, and 3 compared to those in the CTR group (Fig. 1A). The Papp values decreased considerably in a dose-dependent manner on day 3 compared to those in the CTR group (Fig. 1B). These results revealed that ATP promotes the barrier function of 16HBE in a dose-dependent manner.

Real-time PCR analysis revealed that the mRNA levels of E-cadherin, occludin, ZO-2, JAM-A, JAM-C, CLDN1, CLDN3, CLDN4, CLDN6, and CLDN9 on day 3 were considerably higher than those in the CTR group (Fig. 1C). To confirm that this phenomenon was independent of cell proliferation and cell count, we counted the number of viable cells. No significant difference was observed between the viable cell counts in the CTR group and the 500 µM ATP stimulation group on days 0 and 3 (Fig. 1D). These results indicate that ATP promotes airway epithelial barrier formation. Changes in TER values over time differed when ATP stimulation was applied to the apical, basolateral, or bilateral sides, with a significant increase when ATP stimulation was applied to the basolateral and bilateral sides compared to CTR. Consistent with the preferential basolateral localization of P2Y12/P2Y13 in epithelial cells, additional experiments showed that ATP application to the basolateral or bilateral compartments, but not to the apical compartment alone, significantly enhanced TER and reduced FITC–dextran flux in 16HBE14o- monolayers (Fig. 1E).

### Expression of P2X and P2Y receptors in 16 HBE cells

**Fig. 2:**
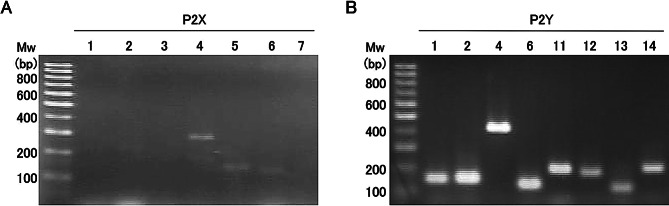
Reverse transcription PCR analysis of P2X and P2Y receptor expression in human airway epithelial cells. Reverse transcription PCR was performed to evaluate the mRNA expression of purinergic receptors in 16HBE cells. (**A**) P2X receptor subtypes (P2 × 1–P2 × 7). (**B**) P2Y receptor subtypes (P2Y1, P2Y2, P2Y4, P2Y6, P2Y12, P2Y13, and P2Y14). The leftmost lane contains the molecular weight markers (100–1,000 bp). Representative results from three independent experiments are shown

The expression of receptors expressed in 16HBE cells was confirmed by reverse transcription PCR to elucidate the signaling pathway involved in barrier function enhancement by ATP. The P2X receptor subtype P2X4 (Fig. 2A) and P2Y receptor subtypes P2Y1, P2Y2, P2Y4, P2Y6, P2Y11, P2Y12, P2Y13, and P2Y14 were expressed in 16HBE cells (Fig. 2B). These results suggest that the P2Y receptor subtypes and P2 × 4 are expressed in 16HBE cells. Although P2 × 4 mRNA was most prominently detected under our experimental conditions, previous studies on 16HBE14o- and related human airway epithelial cells have reported P2 × 4 co-expression with other P2X subtypes such as P2X5 and P2X6, indicating that primer design, culture conditions, and passage history can influence the detectable P2X repertoire [27, 28].

### Stimulation of a selective agonist of P2 receptor promotes epithelial barrier integrity

**Fig. 3:**
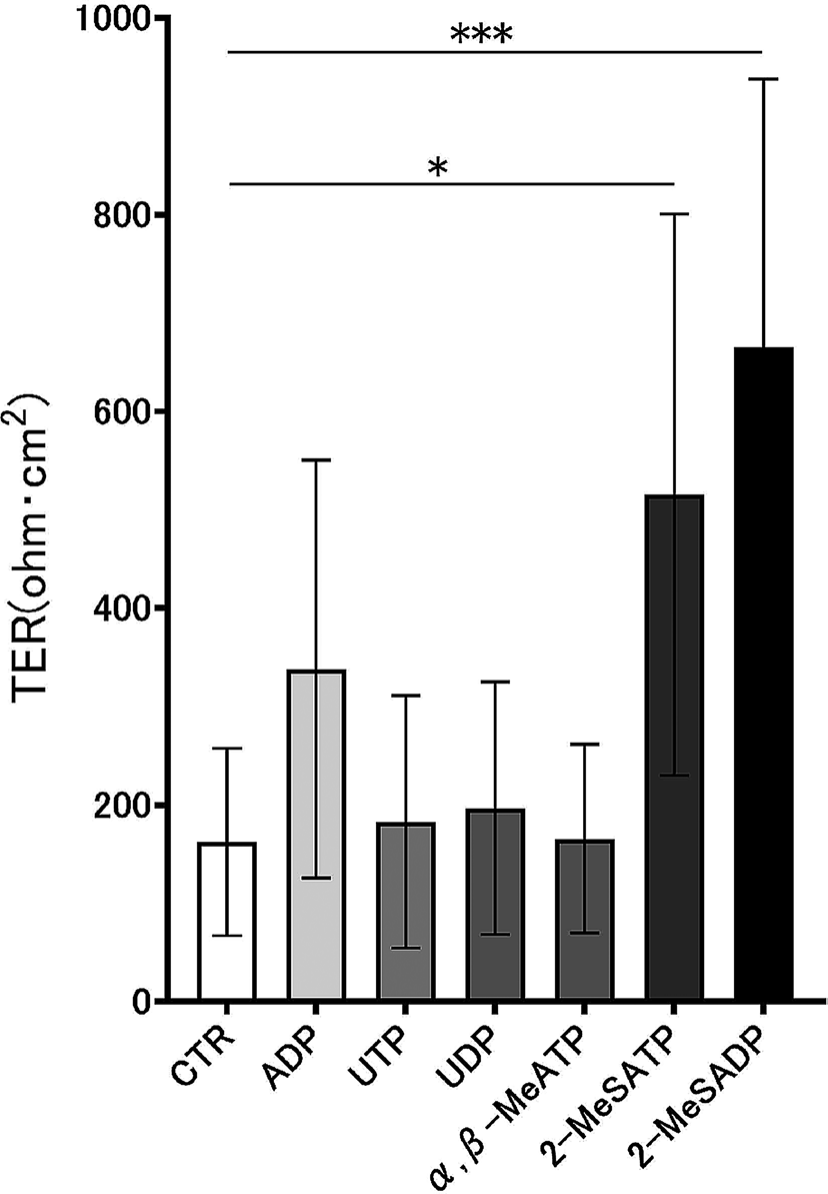
Selective agonists for adenosine triphosphate (ATP) receptors promote barrier function formation on day 3. Examination of the barrier function by αβ-MeATP, 2-MeSATP, adenosine diphosphate (ADP), uridine diphosphate (UDP), uridine triphosphate (UTP), and 2-MeSADP stimulation in 16HBE cells on day 3. Each agonist was applied at a concentration of 500 µM. Transepithelial electrical resistance (TER) was measured upon stimulation with selective agonists on day 3. Results are shown as the means ± standard deviation. Statistical significance was assessed using one-way analysis of variance and multiple comparisons by analysis of variance (*n* = 6). *P < 0.05, ***P < 0.0005

The effects of selective agonists of the P2X and P2Y receptors on TER values were tested to identify the purinergic receptors involved in promoting epithelial barrier formation. The TER values for 2-MeSATP and 2-MeSADP increased significantly on day three compared with CTR (Fig. 3). These results suggest that 2-MeSATP and 2-MeSADP enhance airway epithelial barrier integrity, indicating that P2Y1, P2Y12, and P2Y13 receptors may be involved.

### P2Y receptor-selective antagonists suppress ATP-induced epithelial barrier integrity

**Fig. 4:**
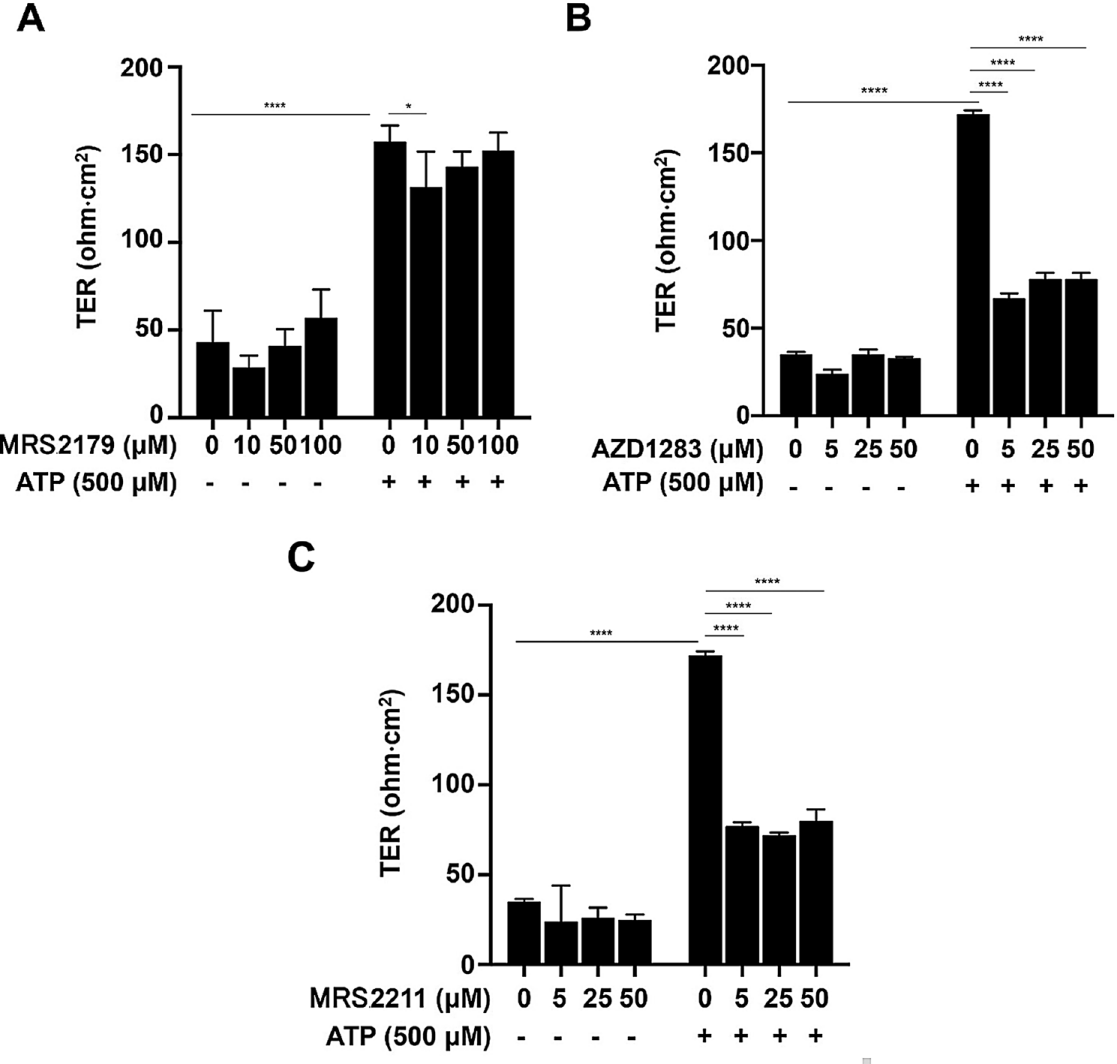
Selective antagonists for adenosine triphosphate (ATP) receptors suppress barrier function formation. The effect of ATP on barrier function following the administration of selective P2 receptor antagonists was investigated. (**A**) Transepithelial electrical resistance (TER) upon ATP stimulation following the administration of MRS2179 (10 µM, 50 µM, and 100 µM). (**B**) TER upon ATP stimulation following the administration of AZD1283 (5 µM, 25 µM, and 50 µM). (**C**) TER upon ATP stimulation following administration of MRS2211 (5 µM, 25 µM, and 50 µM). Results are shown as the means ± standard deviation. Statistical significance was determined by one-way analysis of variance followed by Tukey’s post hoc test. *P < 0.05, ****P < 0.0001. Statistical comparisons were performed between the ATP 0 µM (vehicle control) group and the ATP 500 µM group, and between ATP 500 µM with and without P2Y receptor antagonists (*n* = 6)

These results suggest that P2Y1, P2Y12, and P2Y13 are purinergic receptors that promote epithelial barrier function. To confirm this hypothesis, we used P2Y receptor-selective antagonists. Compared with the ATP alone group, only the 10 µM MRS2179 group had significantly reduced TER values (Fig. 4A). In contrast, ATP-induced TER values considerably decreased in all AZD1283 and MRS2211 concentration groups (Fig. 4B–D). These results suggest that P2Y12 and P2Y13 may be involved in ATP-induced epithelial barrier integrity.

### Involvement of P2Y12 in airway epithelial barrier integrity

**Fig. 5:**
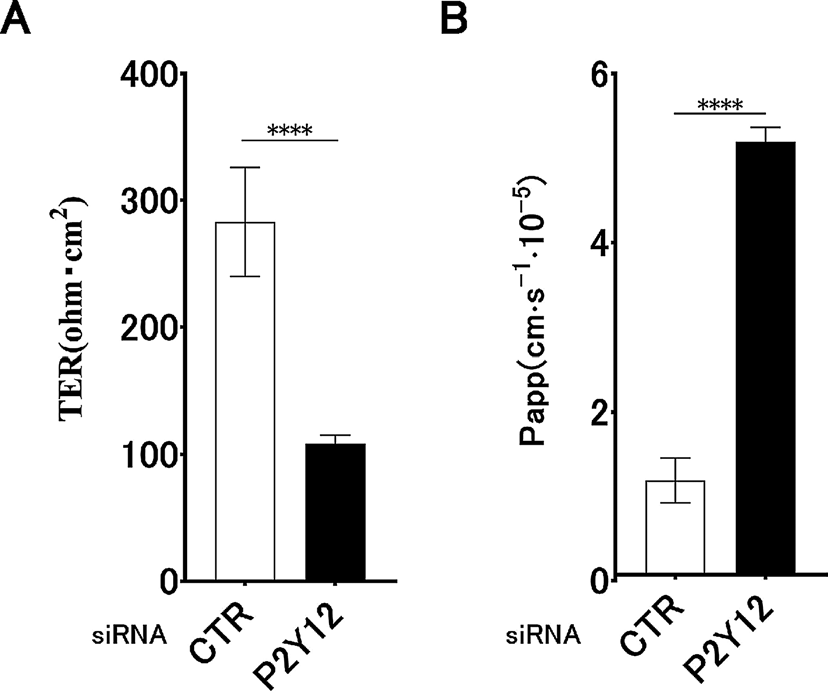
Effects of P2Y12 knockdown on airway epithelial barrier function in 16HBE. Transepithelial electrical resistance (TER) and dextran permeability measurements (Papp) between the P2Y12 knockdown and control (CTR) groups on day 1 post-ATP stimulation. The significant decrease in TER and increased dextran permeability in the P2Y12 knockdown group suggests the potential involvement of P2Y12 in enhancing airway epithelial barrier integrity. Results are expressed as the mean ± SDs, *n* = 3; ****P < 0.0001

Finally, we used specific siRNAs to confirm that P2Y12 is involved in ATP-induced epithelial barrier integrity. We investigated the effects of P2Y12 siRNA knockdown on airway epithelial barrier function using 16HBE cells. Both the P2Y12 knockdown and control groups (CTR) were stimulated with ATP, and TER and dextran permeability were measured on day 1 post-stimulation. Notably, the P2Y12 knockdown group exhibited a significant decrease in TER and a concomitant increase in dextran permeability compared to CTR (Fig. 5). These findings suggest that P2Y12 contributes to ATP-related enhancement of airway epithelial barrier integrity.

## Discussion

This study provides functional evidence suggesting that ATP-related purinergic signaling is associated with enhanced airway epithelial barrier integrity in 16HBE14o- cells and that P2Y12 contributes to this response. Our findings contribute to the growing body of literature that underscores the importance of extracellular ATP as a signaling molecule in regulating epithelial barrier function and homeostasis [16], as well as the importance of extracellular nucleotides such as ATP in modulating essential physiological responses [16]. Notably, our results indicated that ATP-induced promotion of airway epithelial barrier integrity is associated with the upregulation of critical TJ and AJ proteins, including E-cadherin, occludin, ZO-2, JAM-A, JAM-C, and various CLDNs.

The integrity of the airway epithelial barrier is crucial for maintaining homeostasis and defending against external pathogens. Barrier disruption has been implicated in various respiratory diseases, including asthma [4, 6]. This study is significant because it suggests that ATP-related purinergic signaling can enhance airway epithelial barrier integrity in an in vitro airway epithelial model. This is in contrast to previous studies that have primarily focused on the role of ATP in immune responses and inflammation [11, 12, 18].

Our findings provide insight into the molecular mechanisms underlying ATP-related regulation of epithelial barrier integrity. ATP upregulates the expression of crucial TJ and AJ proteins, thereby strengthening the epithelial barrier. This is consistent with previous studies that have reported the attenuation of barrier formation by the TLR3 ligand double-stranded RNA and decreased levels of TJ molecules in 16HBE cells [19]. However, unlike these studies, we found that E-cadherin levels were upregulated, suggesting that ATP has a broader impact on the TJ and AJ structures. This is a significant addition to the existing literature, as it indicates that ATP may be involved in the maintenance and adjustment of the cytoskeleton itself, thereby providing a more comprehensive understanding of its role in epithelial barrier function [20].

Notably, this study identified a specific purinergic receptor, namely P2Y12, as a mediator of ATP-induced airway epithelial barrier integrity enhancement. Previous studies have reported the expression of various P2X and P2Y receptors in airway epithelial cells [18, 21, 22].

To our knowledge, this study provides the first functional evidence suggesting the involvement of P2Y12 in ATP-related regulation of airway epithelial barrier integrity in this in vitro model.

We confirmed P2Y12 involvement using selective antagonists such as AZD1283 and MRS2211, all of which showed a suppressive effect on ATP-induced barrier formation. This is particularly noteworthy because P2Y12 receptors are coupled to the Gi protein of the family of G protein-coupled receptors previously implicated in platelet activation and vascular smooth muscle cell function [23, 24]. These findings may expand our understanding of the physiological roles of these receptors and inform future translational studies.

The localization of these receptors adds another layer of complexity to our understanding. Previous studies have shown that P2Y1, P2Y11, and P2Y12 are only localized to the basolateral membrane in epithelial cells of various tissues [25]. Our study corroborates these findings, as we observed that ATP stimulation from the basolateral side enhanced the airway epithelial barrier integrity more effectively. This observation may have potential implications for how purinergic signaling is accessed in vivo, although further studies will be required before any therapeutic considerations can be made. Accordingly, our experiments further demonstrated that basolateral—but not apical—application of ATP augments TER and decreases FITC–dextran flux in 16HBE14o-monolayers, supporting the concept that ATP- and ADP-dependent signaling at the basolateral surface is particularly important for barrier reinforcement in vivo [13, 18, 26].

Moreover, our study challenges existing paradigms regarding the role of ATP in respiratory diseases. While Idzko et al. reported that extracellular ATP triggers allergic airway inflammation in animal models of asthma [26], our findings indicate that ATP promotes airway epithelial barrier integrity. This apparent contradiction may be reconciled by considering the dual role of ATP in both pro-inflammatory and barrier-enhancing actions, possibly mediated by different receptor subtypes or signaling pathways. ATP promotes the repair of airway epithelial injuries via the epidermal growth factor receptor [27], which has also been implicated in enhancing airway epithelial barrier integrity [28]. These observations support a model wherein sustained, high-amplitude Ca2 + influx through P2 × 7 and related channels promotes junctional disruption and cell death under injurious conditions, whereas more moderate, G protein–coupled P2Y12/P2Y13 signaling can enhance junctional protein expression and cytoskeletal support, thereby stabilizing the barrier under non-injurious or reparative conditions [18, 24, 29].

In addition to ATP, other nucleotides and their metabolites may play roles in modulating the integrity of the airway epithelial barrier. For instance, membrane-type enzymes hydrolyze ATP to generate adenosine, which has been reported to exert anti-inflammatory effects [18]. Furthermore, ATP enhances the formation of the intestinal epithelial barrier [29], suggesting that purinergic signaling may play a broader role in maintaining epithelial barriers throughout the body. Therefore, future studies should aim to elucidate the exact mechanisms whereby purinergic signaling pathways, including both ATP and adenosine, contribute to ATP-induced airway epithelial barrier integrity. As ATP is rapidly hydrolysed by epithelial ectonucleotidases, it is likely that ATP-derived ADP is the predominant agonist at P2Y12 and P2Y13 in our system and that the barrier-enhancing effects we observed are indicative of an ATP→ADP→P2Y12/P2Y13 purinergic signaling pathway; future studies using apyrase, hydrolysis-resistant ATP analogues, and direct ADP stimulation will be required to provide more clarification on these findings [18, 20, 24].

This study has several limitations. First, our findings were based solely on immortalized human airway epithelial cell lines, which may not fully reflect in vivo conditions. Second, the evaluation of AJ and TJ proteins was primarily based on mRNA levels without comprehensive protein localization data. Third, mechanistic insights into receptor localization and ATP release dynamics remain unresolved. In addition, although we discussed the potential contribution of ATP-derived ADP to P2Y12/P2Y13 activation, we did not directly dissect the relative contributions of ATP versus ADP in our experimental system. Future studies using primary epithelial cells, in vivo models, and advanced imaging techniques are warranted. Furthermore, we did not assess P2Y12 protein expression or subcellular localization in 16HBE14o- cells, and we did not directly examine the contribution of P2Y13 using gene-silencing approaches; future studies using immunofluorescence or western blotting in both cell lines and primary human airway epithelia, together with receptor-specific knockdown or gene editing, are therefore required to corroborate and extend our mRNA-based findings [17, 27].

## Conclusion

This study provides functional evidence that ATP-related purinergic signaling via P2Y12 contributes to airway epithelial barrier integrity in vitro. Further studies using primary human airway epithelial cells, protein-level analyses, and in vivo models are required to determine the physiological and clinical relevance of these findings.

## Data Availability

The datasets used and/or analyzed during the current study are available from the corresponding author on reasonable request.

## Declarations

### Authors’ information

KT, SM, SO, SS, MU, SY, YKu, KM, and YKo are with the Division of Respiratory Medicine, Department of Internal Medicine, Nihon University School of Medicine, Tokyo, Japan. YG is a Professor in the same division.

## Abbreviations

ADP: Adenosine diphosphate
AJ: Adherens junction
ATP: Adenosine triphosphate
cDNA: Complementary DNA
CLDN: Claudin
CTR: Control
EGFR: Epidermal growth factor receptor
FITC: Fluorescein isothiocyanate
HDM: House dust mite allergens
MEM: Minimum essential medium
OVA: Ovalbumin
Papp: Permeability coefficient
siRNA: Short-interfering RNA
TER: Transepithelial electrical resistance
TJ: Tight junction
UDP: Uridine diphosphate
UTP: Uridine triphosphate

## References

[1] Schleimer RP, Kato A, Kern R, Kuperman D, Avila PC, Epithelium. At the interface of innate and adaptive immune responses. J Allergy Clin Immunol. 2007;120(6):1279–84. 17949801 10.1016/j.jaci.2007.08.046 PMC2810155

[2] Shen L, Weber CR, Raleigh DR, Yu D, Turner JR. Tight junction pore and leak pathways: A dynamic duo. Annu Rev Physiol. 2011;73(1):283–309. 20936941 10.1146/annurev-physiol-012110-142150 PMC4655434

[3] Rusu AD, Georgiou M. The multifarious regulation of the apical junctional complex. Open Biol. 2020;10(2):190278. 32070233 10.1098/rsob.190278 PMC7058937

[4] Loxham M, Davies DE. Phenotypic and genetic aspects of epithelial barrier function in asthmatic patients. J Allergy Clin Immunol. 2017;139(6):1736–51. 28583446 10.1016/j.jaci.2017.04.005 PMC5457128

[5] Xiao C, Puddicombe SM, Field S, Haywood J, Broughton-Head V, Puxeddu I, et al. Defective epithelial barrier function in asthma. J Allergy Clin Immunol. 2011;128(3):549–e56512. 21752437 10.1016/j.jaci.2011.05.038

[6] Holgate ST. Epithelium dysfunction in asthma. J Allergy Clin Immunol. 2007;120(6):1233–44. quiz 1245–6. 18073119 10.1016/j.jaci.2007.10.025

[7] de Boer WId, Sharma HS, Baelemans SMI, Hoogsteden HC, Lambrecht BN, Braunstahl GJ. Altered expression of epithelial junctional proteins in atopic asthma: Possible role in inflammation. Can J Physiol Pharmacol. 2008;86(3):105–12. 18418437 10.1139/y08-004

[8] Ford ES. The epidemiology of obesity and asthma. J Allergy Clin Immunol. 2005;115(5):897–909. quiz 910. 15867841 10.1016/j.jaci.2004.11.050

[9] Hammad H, Chieppa M, Perros F, Willart MA, Germain RN, Lambrecht BN. House dust mite allergen induces asthma via toll-like receptor 4 triggering of airway structural cells. Nat Med. 2009;15(4):410–6. 19330007 10.1038/nm.1946 PMC2789255

[10] Hammad H, Lambrecht BN. Barrier epithelial cells and the control of Type 2 immunity. Immunity. 2015;43(1):29–40. 26200011 10.1016/j.immuni.2015.07.007

[11] Faas MM, Sáez T, de Vos P. Extracellular ATP and adenosine: The Yin and Yang in immune responses? Mol Aspects Med. 2017;55:9–19. 28093236 10.1016/j.mam.2017.01.002

[12] Müller T, Robaye B, Vieira RP, Ferrari D, Grimm M, Jakob T, et al. The purinergic receptor P2Y2 receptor mediates chemotaxis of dendritic cells and eosinophils in allergic lung inflammation. Allergy. 2010;65(12):1545–53. 20880147 10.1111/j.1398-9995.2010.02426.x

[13] Okada SF, Nicholas RA, Kreda SM, Lazarowski ER, Boucher RC. Physiological regulation of ATP release at the apical surface of human airway epithelia. J Biol Chem. 2006;281(32):22992–3002. 16754672 10.1074/jbc.M603019200 PMC2924190

[14] Button B, Okada SF, Frederick CB, Thelin WR, Boucher RC. Mechanosensitive ATP release maintains proper mucus hydration of airways. Sci Signal. 2013;6(279):ra46. 23757023 10.1126/scisignal.2003755 PMC3791865

[15] Wesley UV, Bove PF, Hristova M, McCarthy S, van der Vliet A. Airway epithelial cell migration and wound repair by ATP-mediated activation of dual oxidase 1. J Biol Chem. 2007;282(5):3213–20. 17135261 10.1074/jbc.M606533200

[16] Mori T, Shiratsuchi N, Sato N, Chaya A, Tanimura N, Ishikawa S, et al. Extracellular ATP facilitates cell extrusion from epithelial layers mediated by cell competition or apoptosis. Curr Biol. 2022;32(10):2144–e21595. 35417667 10.1016/j.cub.2022.03.057

[17] Jacobson KA, Müller CE. Medicinal chemistry of adenosine, P2Y and P2X receptors. Neuropharmacology. 2016;104:31–49. 26686393 10.1016/j.neuropharm.2015.12.001 PMC4871727

[18] Idzko M, Ferrari D, Eltzschig HK. Nucleotide signalling during inflammation. Nature. 2014;509(7500):310–7. 24828189 10.1038/nature13085 PMC4222675

[19] Gon Y, Maruoka S, Kishi H, Kozu Y, Kuroda K, Mizumura K, et al. DsRNA disrupts airway epithelial barrier integrity through down-regulation of claudin members. Allergol Int. 2016;65(Suppl):S56–8. 27238378 10.1016/j.alit.2016.04.006

[20] Lazarowski ER, Tarran R, Grubb BR, van Heusden CA, Okada S, Boucher RC. Nucleotide release provides a mechanism for airway surface liquid homeostasis. J Biol Chem. 2004;279(35):36855–64. 15210701 10.1074/jbc.M405367200 PMC2943374

[21] Jacobson KA, Balasubramanian R, Deflorian F, Gao ZG. G protein-coupled adenosine (P1) and P2Y receptors: Ligand design and receptor interactions. Purinergic Signal. 2012;8(3):419–36. 22371149 10.1007/s11302-012-9294-7 PMC3360101

[22] Abbracchio MP, Burnstock G, Boeynaems JM, Barnard EA, Boyer JL, Kennedy C, et al. International Union of Pharmacology LVIII: Update on the P2Y G protein-coupled nucleotide receptors: From molecular mechanisms and pathophysiology to therapy. Pharmacol Rev. 2006;58(3):281–341. 16968944 10.1124/pr.58.3.3 PMC3471216

[23] von Kügelgen I. Pharmacological profiles of cloned mammalian P2Y-receptor subtypes. Pharmacol Ther. 2006;110(3):415–32. 16257449 10.1016/j.pharmthera.2005.08.014

[24] von Kügelgen I, Hoffmann K. Pharmacology and structure of P2Y receptors. Neuropharmacology. 2016;104:50–61. 26519900 10.1016/j.neuropharm.2015.10.030

[25] Laidlaw TM, Cahill KN, Cardet JC, Murphy K, Cui J, Dioneda B, et al. A trial of type 12 purinergic (P2Y12) receptor inhibition with prasugrel identifies a potentially distinct endotype of patients with aspirin-exacerbated respiratory disease. J Allergy Clin Immunol. 2019;143(1):316–e247. 29890239 10.1016/j.jaci.2018.06.001 PMC6286686

[26] Idzko M, Hammad H, van Nimwegen M, Kool M, Willart MAM, Muskens F, et al. Extracellular ATP triggers and maintains asthmatic airway inflammation by activating dendritic cells. Nat Med. 2007;13(8):913–9. 17632526 10.1038/nm1617

[27] Wolff SC, Qi AD, Harden TK, Nicholas RA. Polarized expression of human P2Y receptors in epithelial cells from kidney, lung, and colon. Am J Physiol Cell Physiol. 2005;288(3):C624–32. 15525684 10.1152/ajpcell.00338.2004

[28] Sarojini H, Billeter AT, Eichenberger S, Druen D, Barnett R, Gardner SA, et al. Rapid tissue regeneration induced by intracellular ATP delivery - A preliminary mechanistic study. PLoS ONE. 2017;12(4):e0174899. 28380006 10.1371/journal.pone.0174899 PMC5381896

[29] Crosby LM, Waters CM. Epithelial repair mechanisms in the lung. Am J Physiol Lung Cell Mol Physiol. 2010;298(6):L715–31. 20363851 10.1152/ajplung.00361.2009 PMC2886606

